# Causal relationship between circulating insulin-like growth factor-1 and Parkinson’s disease: a two-sample Mendelian randomization study

**DOI:** 10.3389/fnagi.2024.1333289

**Published:** 2024-04-17

**Authors:** Jiahao Xu, Peidong Fan, Jiacheng Yang, Mingjuan Yin, Jun Wu, Chao Chen, Jindong Ni

**Affiliations:** ^1^Department of Epidemiology and Health Statistics, School of Public Health, Guangdong Medical University, Dongguan, China; ^2^Department of Neurology, Second Hospital Affiliated of Xinjiang Medical University, Xinjiang, China; ^3^Maternal and Child Research Institute, Shunde Women and Children’s Hospital, Guangdong Medical University, Foshan, China; ^4^Precision Laboratory, School of Public Health, Guangdong Medical University, Dongguan, China

**Keywords:** Parkinson’s disease, causality, genetics, circulating insulin-like growth factor-1, Mendelian randomization

## Abstract

**Background:**

Linear associations between circulating insulin-like growth factor-1 (IGF-1) levels and Parkinson’s disease (PD) have been evidenced in observational studies. Yet, the causal relationship between IGF-1 levels and PD remains obscure. We conducted Mendelian randomization to examine the correlation between genetically predicted IGF-1 levels and PD.

**Methods:**

By reviewing genome-wide association studies (GWAS) that are publicly accessible, we uncovered SNPs linked to both serum concentrations of IGF-1 and PD. A two-sample Mendelian randomization (MR) analysis was carried out to evaluate the individual effect of IGF-1 on PD.

**Results:**

In a primary causal effects model in MR analysis, employing the inverse-variance weighted (IVW) method, IGF-1 levels exhibited a notable association with the risk of PD (OR, 1.020, 95% CI, 1.003–1.038, *p* = 0.0215). Multiple evaluations revealed that horizontal pleiotropy was improbable to distort the main results (MR-Egger: P PD intercept =0.719), and no bias was detected by leave-one-out analysis.

**Conclusion:**

This study unearthed evidence indicating that heightened IGF-1 levels might be causally correlated with an increased risk of PD.

## 1 Introduction

Research into human aging has intensified, and the investigations into brain health have become more in-depth. Parkinson’s disease (PD) is predominantly an age-associated condition. Although PD presents in a minor percentage of younger adults, the majority of patients (over 75%) are over the age of 65 (Pringsheim et al., 2014; Deuschl et al., 2020). PD is a neurodegenerative disease attributed to progressive neuronal loss in the substantia nigra pars compacta, with typical clinical features including tremor, rigidity, bradykinesia/akinesia, and postural instability (Scorza et al., 2021). Moreover, PD, inflicting a considerable societal and economic burden worldwide, serves as a principal cause of disability and mortality among the elderly (Heemels, 2016; Berson et al., 2018). In clinical practice, there are no established curative methods for PD, and age stands as the sole universally accepted risk factor (Dorsey et al., 2007; Kalia and Lang, 2015; Lee and Gilbert, 2016; Scorza et al., 2018; Balestrino and Schapira, 2020).

In recent years, circulating insulin-like growth factor-1 (IGF-1), a neurotrophic hormone integral to systemic development, has gained increasing recognition for its pivotal role in brain health, influencing both central nervous system plasticity and the development of all major neural cells (O’Kusky and Ye, 2012; Dyer et al., 2016).

In a recent investigation, a linkage between IGF-1 and PD risk has been unveiled (Cao et al., 2023). It explored and fully characterized the dose–response correlation between IGF-1 and PD risk within a sizable cohort of the general populace. Nonetheless, owing to the propensity for observational inquiries to incur confounding biases, such as the potential impact of diverse underlying maladies on the varying levels of IGF-1, novel methodologies are imperative for scrutinizing causal relationships.

Mendelian randomization (MR) is a technique that is used to infer causality by employing one or more genetic variants that influence a risk factor. These genetic tools help discern the impact of the risk factor on the disease (Davey Smith and Hemani, 2014). We used an MR design to explore the causal relationship between IGF-1 and PD risk.

## 2 Materials and methods

### 2.1 Study design

**Figure 1:**
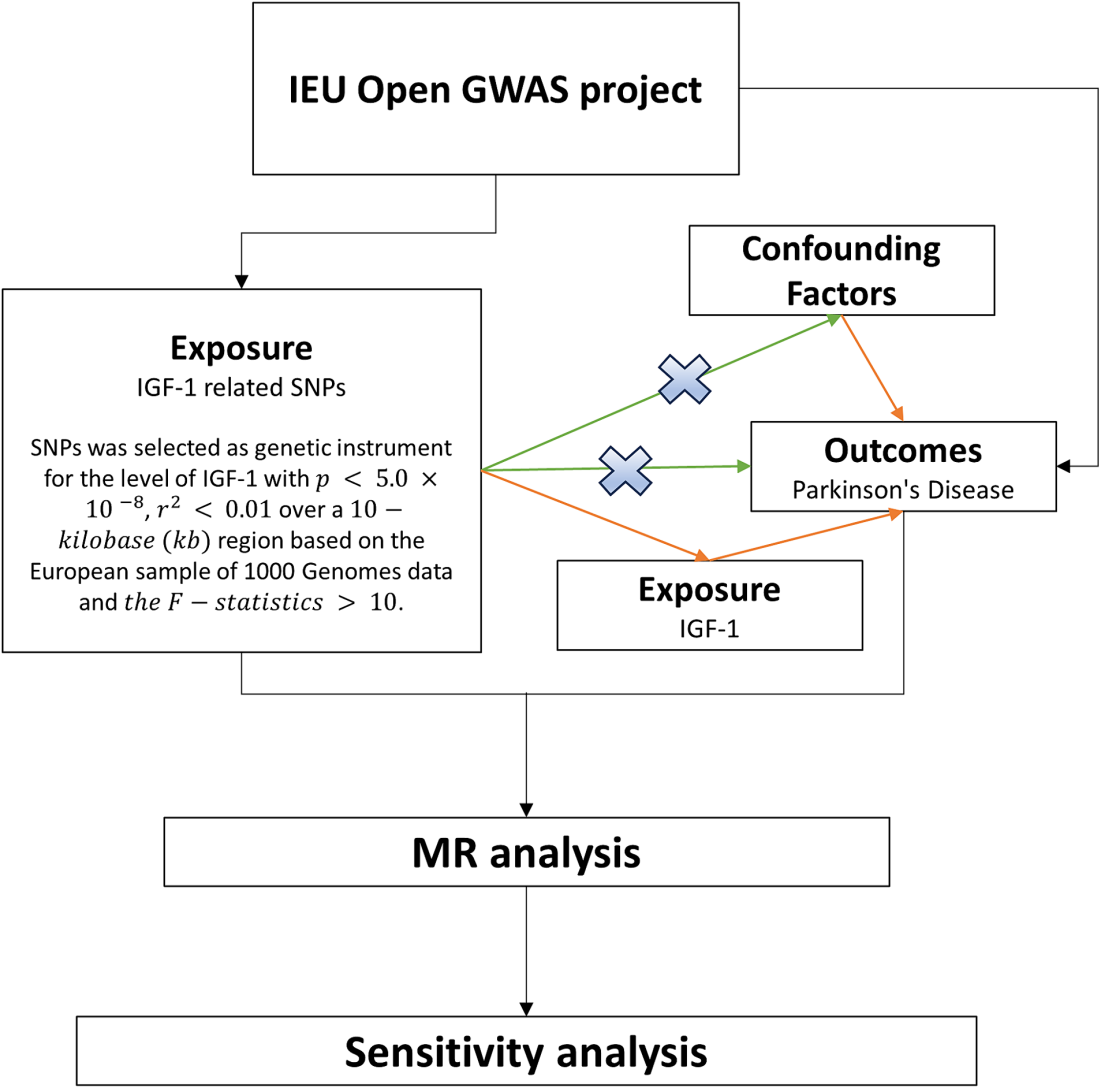
Flowchart of MR in this study. The study data were from the IEU Open GWAS project, where SNPs for exposure and outcome were first extracted, and instrumental variables were screened based on the SNPs for exposure. The causal effect was further explored using two-sample Mendelian randomization, and sensitivity analyses were used to test the stability of the results. MR, Mendelian randomization; SNP, Single Nucleotide Polymorphisms; IEU, Integrative Epidemiology Unit; GWAS, Genome-Wide Association Studies; IGF-1, Insulin-like growth factor-1.

MR analysis, harnessing genetic allocation variation, remains impervious to the influence of confounding factors, thereby serving as a surrogate for risk factors in instrumental variable analyses. Genetic variation is deemed effective only when closely correlated with relevant risk factors and influences outcomes solely through exposure, rather than directly affecting results (Smith and Ebrahim, 2003). Considering the susceptibility of observational studies to the impact of reverse causality and undiscovered confounding factors, this study employed a two-sample MR analysis to explore the causal relationship between circulating IGF-1 levels and PD. The SNP data for IGF-1 and PD were sourced from the Genome-Wide Association Studies (GWAS) database (Figure 1).

### 2.2 Genetic instrument selection

The data for IGF-1 were obtained from 358,072 European-descent participants in the UK Biobank, with a sample size of 342,439 for IGF-1. We satisfied the conditions for SNPs to serve as effective instrumental variables (IVs): (i) strong correlation with exposure ( $p<5.0\times 10^{-8}$ ); (ii) SNP independence ensured by $r^{2}<0.01$ over a 10-kilobase (kb) region based on European samples from the 1,000 Genomes data; (iii) avoidance of pathways where SNPs could directly influence outcomes (horizontal pleiotropy). Weak IV bias was mitigated using an F-statistic >10 (Huang et al., 2021). As a result, 574 SNPs were selected from 13,586,000 available SNPs as IVs for circulating IGF-1 levels. Data were downloaded from Integrative Epidemiology Unit (IEU) GWAS database (see Table 1).

**Table 1:** Data sources for exposure and outcome.

	Samples size (case/control)	GWAS ID	Author	Consortium	SNPs available
IGF-1	342,439	ukb-d-30770	Neale lab	NA	13,586,000
PD	482,730 (33,674/449,056)	ieu-b-7	Nalls MA	International Parkinson’s Disease Genomics Consortium	17,891,936

### 2.3 Data origin of PD

Summary-level data for PD (GWAS ID: ieu-b-7) were sourced from the International Parkinson’s Disease Genomics Consortium, comprising 33,674 cases among a sample size of 482,730. The aforementioned data can be retrieved through the OpenGWAS project (mrcieu.ac.uk) and are summarized in Table 1.

### 2.4 Statistical analysis

In models devoid of horizontal pleiotropy, IVW yields results of maximal efficiency, rendering it as the reference in this study employing a random-effects model (Burgess et al., 2016; Burgess and Thompson, 2017; Davies et al., 2019). Several other approaches were utilized to ascertain the consistency of results. These include the simple median method, which provides reliable estimates even in the presence of at least 50% non-effective IVs; the weighted median method, which, building upon this premise, accounts for differences in estimation accuracy; and the maximum likelihood method, characterized by its smaller standard errors, used to obtain parameters of the probability density function for the database (Milligan, 2003; Burgess et al., 2017).

Furthermore, to ascertain potential impacts on MR outcomes and eliminate violations of MR assumptions due to heterogeneity and pleiotropy within the utilized IVs, a comprehensive assessment was conducted. This included heterogeneity, pleiotropy, and sensitivity, corresponding to Egger regression combined with MR-PRESSO global tests, Cochran’s Q statistic, and single SNP analysis combined with leave-one-out sensitivity analysis, respectively. Egger regression analysis and MR-PRESSO global tests were deemed to evaluate potential directional pleiotropy (Verbanck et al., 2018; Ong and MacGregor, 2019). If directional pleiotropy was detected with a *p*-value <0.05 based on the intercept from MR-Egger, MR-PRESSO was utilized for secondary assessment, followed by outlier removal and subsequent recalculation of causal effects. Cochran’s Q statistic from IVW and MR-Egger was utilized to examine the magnitude of heterogeneity (Storey and Tibshirani, 2003). MR-PRESSO was also utilized to test for it. Single SNP analysis and leave-one-out sensitivity analysis were utilized to determine if individual SNPs were sufficient to impact overall results. In leave-one-out plots, the harmonious distribution of all lines around 0 indicated that results were unaffected by which SNP was removed.

When *p* < 0.05 with two-tailed testing, the results are considered significant. All primary outcomes were obtained using the “TwoSampleMR” package within the R (version 4.1.2) environment.

## 3 Results

**Figure 2:**
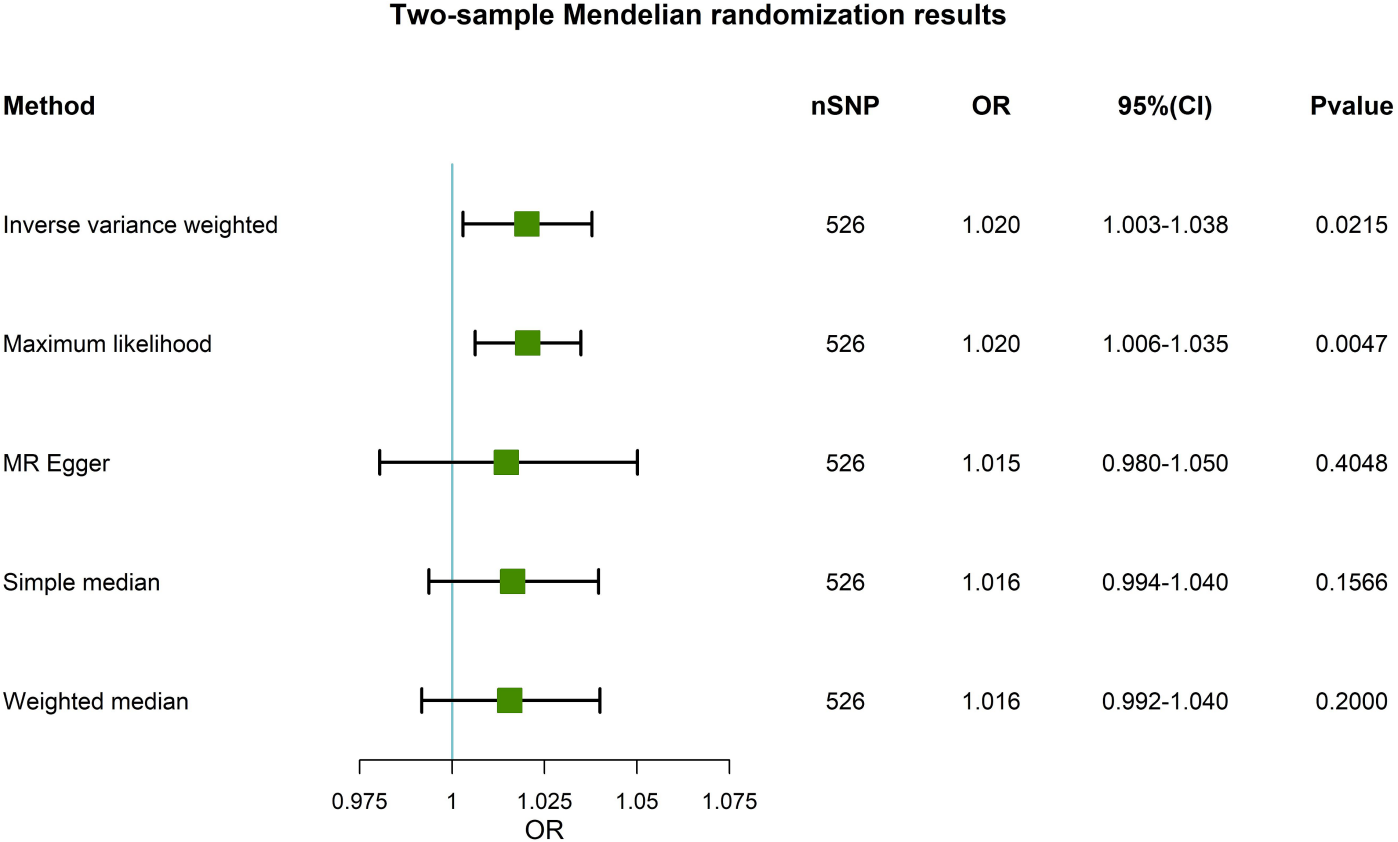
A forest plot showing the association of genetically predicted circulating IGF-1 with PD. Five Mendelian randomization methods are used. nSNP is the number of instrumental variables selected for MR. OR is the PD risk ratio corresponding to each unit increase in circulating IGF-1 levels. When the OR (95% CI) is greater than 1, it means that the PD risk increases. On the contrary, the risk of PD is reduced. A *p* value less than 0.05 indicates statistical significance between circulating IGF-1 levels and PD risk. MR, Mendelian randomization; OR, Odds Ratio; CI, Confidence Interval; SNP, Single Nucleotide Polymorphisms; PD, Parkinson’s disease; IGF-1, Insulin-like growth factor-1.

On the basis of a primary causal effects model with MR analyses through the IVW method, IGF-1 was significantly associated with the risk of PD (OR, 1.020; 95% CI, 1.003–1.038, *p* = 0.0215, Figure 2). The maximum likelihood results were consistent with IVW (OR, 1.020, 95% CI, 1.006–1.035, *p* = 0.0047). The directions of MR-Egger, weighted median, and simple median results were exactly in line with the estimates of IVW revealing significant robustness of the main analysis.

There might be heterogeneity ( $P_{IVW}<0.001;P_{MR\;\mathrm{PRESSO}}<0.001;P_{MR\;\mathrm{Egger}}<0.001$ , Table 2). Regardless, the IVW estimate remained unbiased, affirming the conclusion’s reliability and acceptability. In addition, despite some contradiction noted in the horizontal pleiotropy assessment between MR results (*p* = 0.719) and MR-PRESSO, the uniformity in results (OR, 1.020; 95% CI, 1.004–1.036, *p* = 0.0140, Table 3) persisted across outlier-corrected analysis in MR-PRESSO.

**Table 2:** Heterogeneity and pleiotropy test results.

Disease	Heterogeneity *p* value		Pleiotropy *p* value	
	IVW	MR egger	MR-PRESSO	Egger intercept
Parkinson’s disease	<0.001	<0.001	<0.001	0.719

**Table 3:** MR-PRESSO outlier-corrected analysis for PD.

Outcomes	Exposure	MR-PRESSO	Number of outliers	OR (95% CI)	*p*-value
Parkinson’s disease	IGF-1	Raw	/	1.020 (1.003–1.038)	0.0219
		Outlier-corrected	4	1.020 (1.004–1.036)	0.0140

**Figure 3:**
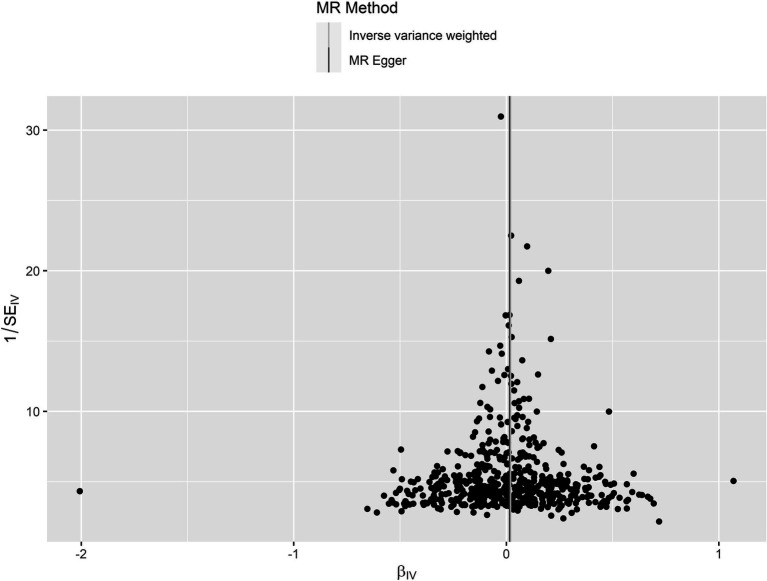
A funnel plot for circulating IGF-1 on PD. The figure shows that the scatter distribution exhibits a symmetrical shape with essentially no deviation from the overall. SE, Standard Error; MR, Mendelian randomization.

**Figure 4:**
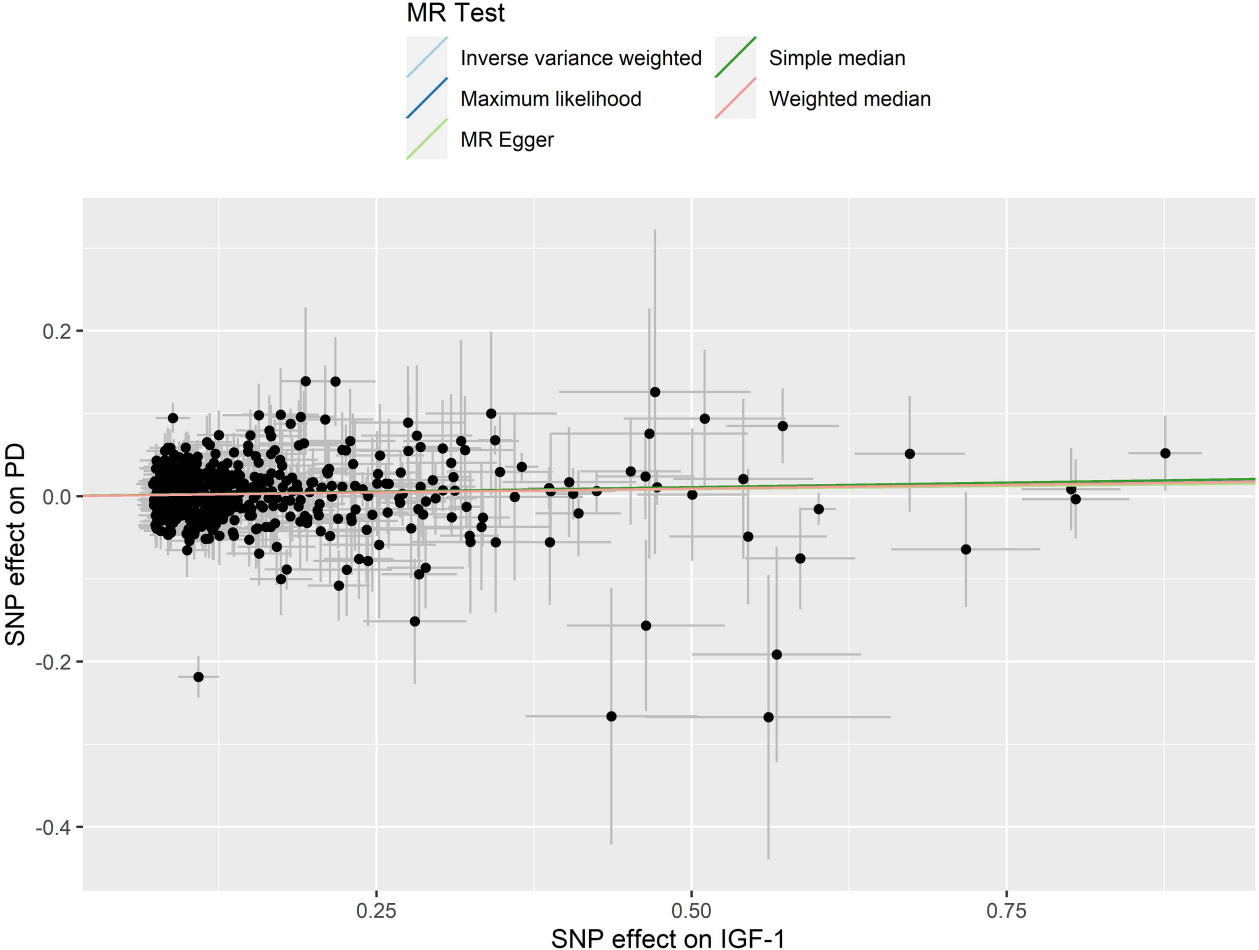
A scatter plot for circulating IGF-1 on PD. The figure showing the genetic correlation between circulating IGF-1 levels and PD risk based on different MR methods. The slopes of the lines represent the causal effect of each method. The slope of the straight line is greater than 0, indicating a positive overall effect of circulating IGF-1 levels on PD risk. SNP, Single Nucleotide Polymorphisms; IGF-1, Insulin-like growth factor-1; PD, Parkinson’s disease.

The leave-one-out analysis guarantees the resilience, as no individual SNP exhibits a significant impact (Supplementary Figure S1). Furthermore, the symmetry observed in the funnel plot (Figure 3) indicates the satisfactory fulfillment of the INSIDE assumption (Bowden et al., 2015). A scatter plot is presented in Figure 4, which shows consistency in the results.

## 4 Discussion

Within our investigation, leveraging GWAS data from those of European ancestry, two independent MR analyses unveil a causal association between IGF-1 and PD risk to a degree. The robust findings and thorough sensitivity analyses adeptly validate the fulfilment of the three fundamental assumptions: relevance, independence, and exclusion restriction assumption.

Substantial evidence emphasizes the notable association between IGF-1 and brain health, particularly in conditions such as major depressive disorder (MDD), anxiety, and cognitive impairments (Mosiołek et al., 2021; Shi et al., 2023). Degenerative neurological conditions with greater impacts on populations have been linked to IGF-1 alterations (Watanabe et al., 2005; Gasperi and Castellano, 2010; Ghazi Sherbaf et al., 2018; Gubbi et al., 2018). However, the findings remain inconclusive when it comes to PD development. While both basic and translational research consistently assert that IGF-1 provides direct protection to all neuronal cells and can inhibit inflammatory cascade reactions at the cellular level (Fernandez et al., 2012; Labandeira-Garcia et al., 2017), there is currently a dearth of scholarly discourse addressing whether this anti-inflammatory effect holds protective implications in animal experiments. Furthermore, compelling evidence has linked IGF-1 to the initiation and progression of PD (Castilla-Cortázar et al., 2020).

In a study using the UK Biobank, the male sex was identified as a primary risk factor for PD, with elevated levels of IGF-1 ranking closely behind. This underscored its significant role in the patho-mechanisms of PD and its potential as a predictive biomarker (Allwright et al., 2023).

Elevated serum IGF-1 levels in PD patients have been negatively correlated with nonmotor symptoms, such as anxiety, depression, and cognitive dysfunction, and combining IGF-1 with EGF enhances the diagnostic value for PD (Shi et al., 2023).

A prospective study confirmed a positive association between IGF-1 concentration and the risk of PD (Cao et al., 2023), which is consistent with the direction of effect results obtained from our MR analysis. This association may be due to the involvement of IGF-1 in nerve growth and formation (Rabinovsky, 2004). IGF-1 serum concentrations might become an important biomarker for assessing the risk of PD, providing new perspectives on the prevention of PD.

The study had several strengths and limitations. The main strength was that Mendelian randomization analysis enhanced the comprehensive assessment of the association between circulating IGF-1 and PD by reducing bias from residual confounding and reverse causation. Moreover, the study used a large amount of PD case–control data, which was sufficient to detect even small effects. In addition, the genetic tools for IGF-1 had good validity, which ensured the robustness of our results. Despite the strengths of this study, there were some weaknesses. Firstly, the implementation of sex-specific MR analysis serves to address the inquiry stemming from the sex correlation of growth hormone-stimulated IGF-1 (Brabant and Wallaschofski, 2007). Nevertheless, despite exhaustive online searches, suitable data sources for analysis is elusive. Hence, presently, the initiation of hormone-related gender-stratified GWAS and subsequent core MR analysis were warranted. Secondly, we encounter a conspicuous limitation whereby the population is exclusively of European descent, hence precluding generalizability to other ethnicities, thereby rendering the applicability of our findings uncertain when extrapolated to different populations. Finally, the potential for a non-linear relationship between them cannot be disregarded, even though prevailing research indicates a linear relationship, necessitating analysis within the context of clinical practice.

## 5 Conclusion

This MR analysis report corroborates a causal nexus between serum IGF-1 levels and PD, establishing a pivotal groundwork for the forthcoming clinical diagnosis and management of PD.

## Data availability statement

The datasets presented in this study can be found in online repositories. The names of the repository/repositories and accession number(s) can be found in the article/Supplementary material.

## Ethics statement

Ethical approval was not required for the study involving humans in accordance with the local legislation and institutional requirements. Written informed consent to participate in this study was not required from the participants or the participants’ legal guardians/next of kin in accordance with the national legislation and the institutional requirements.

## Author contributions

JX: Writing – original draft, Writing – review & editing. PF: Conceptualization, Methodology, Writing – review & editing. JY: Writing – original draft. MY: Validation, Writing – review & editing. JW: Conceptualization, Writing – review & editing. CC: Methodology, Writing – review & editing. JN: Conceptualization, Writing – review & editing.
