# Supplementary figures and images for: Causal relationship between circulating insulin-like growth factor-1 and Parkinson’s disease: a two-sample Mendelian randomization study
Source: Front Aging Neurosci. 2024 Apr 17;16:1333289. doi: 10.3389/fnagi.2024.1333289 (PMC11064709; doi:10.3389/fnagi.2024.1333289)

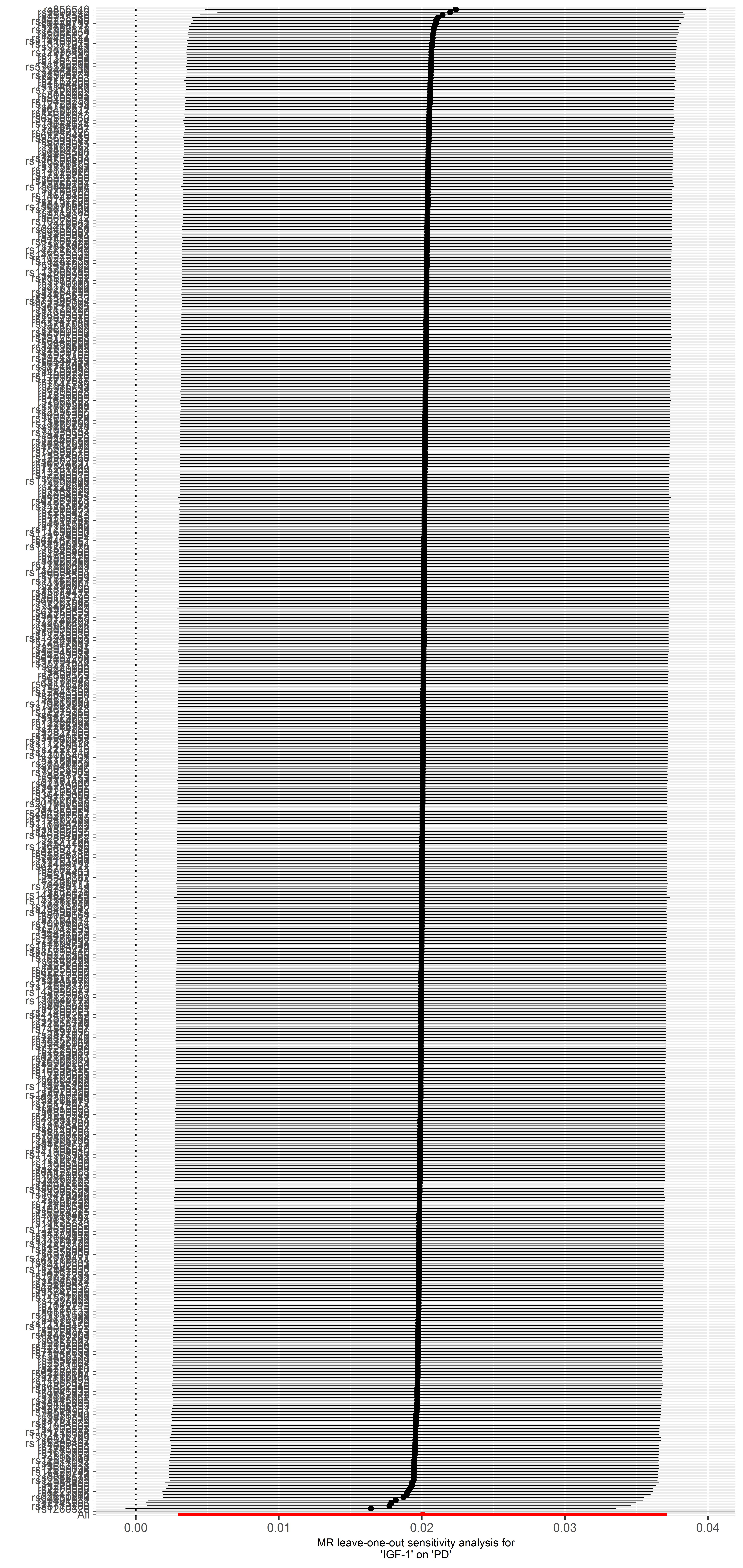

Supplement: Supplementary file 2 [file Image_1.JPEG]
